# Isolated Septal Branch Occlusion Causing Septal Myocardial Infarction, With Suspected Embolic Etiology From Left Atrial Appendage Thrombus: A Case Report

**DOI:** 10.1155/cric/6815054

**Published:** 2026-03-11

**Authors:** A. Jano, A. Hamadanchi, A. Große, P. C. Schulze, R. Surber

**Affiliations:** ^1^ Department of Cardiology, Angiology, and Intensive Care Unit, Universitätsklinikum Jena, Jena, Thuringia, Germany, uniklinikum-jena.de

**Keywords:** anticoagulation therapy, atrial fibrillation, left atrial appendage, septal myocardial infarction, thrombus

## Abstract

We present a case of a 76‐year‐old female who experienced an isolated embolic occlusion of the septal branch, resulting in a septal myocardial infarction (MI) due to a thrombus in the left atrial appendage (LAA). The patient′s history included atrial fibrillation (AF), multiple allergies, and a self‐discontinued anticoagulation regimen. This case underscores the importance of anticoagulation therapy in AF patients and highlights the challenges of managing patients with complex medical histories and medication intolerances.

## 1. Introduction

Thrombosis and subsequent embolic events are well‐documented complications of atrial fibrillation (AF), often leading to ischemic strokes or systemic emboli [1]. The risk of stroke in AF patients is significantly elevated, with studies indicating that AF increases the risk of ischemic stroke by fivefold [2]. The left atrial appendage (LAA) is the primary site of thrombus formation in patients with nonvalvular AF, contributing to approximately 90% of left atrial thrombi [3]. Consequently, anticoagulation therapy is a cornerstone in the management of AF to prevent thromboembolic complications [4].

However, isolated thrombotic occlusion of the septal branch resulting in a septal myocardial infarction is a rare and underreported phenomenon. The septal branches of the coronary arteries supply the interventricular septum, and their occlusion can lead to a localized infarction [5]. The pathophysiology behind such an isolated event often involves embolic sources such as LAA thrombi, which can travel and lodge in smaller coronary vessels [6].

Despite the well‐established benefits of anticoagulation, patient adherence remains a significant challenge. Intolerance to anticoagulant medications, whether due to side effects or perceived quality‐of‐life impacts, often leads patients to discontinue therapy [7]. This case report details the clinical presentation, diagnostic evaluation, and management of an elderly female patient with known AF who experienced an isolated thrombotic occlusion of the septal branch, leading to a septal myocardial infarction due to an LAA thrombus. This case underscores the importance of patient education and adherence to medical recommendations in managing AF.

## 2. Case Presentation

A 76‐year‐old female presented with right arm weakness and pain radiating to the chest. The symptoms prompted her to call emergency services. Her medical history was significant for AF diagnosed in 2019, multiple allergies (penicillin, doxycycline, indomethacin, histamine, and egg protein), and intolerance to nebivolol and apixaban, which led her to discontinue all medications and rely on natural remedies. She reported feeling better with a heart rate above 100 bpm and experienced lethargy and fatigue with a lower heart rate.

In June 2024, the patient consulted her cardiologist and was informed about the risks of stroke and death associated with untreated AF. Despite this, she opted against resuming her medication. An echocardiogram at that time revealed severe tricuspid regurgitation (TI) Grade III and an enlarged right ventricle with elevated systolic pulmonary artery pressure (sPAP).

**Figure 1:**
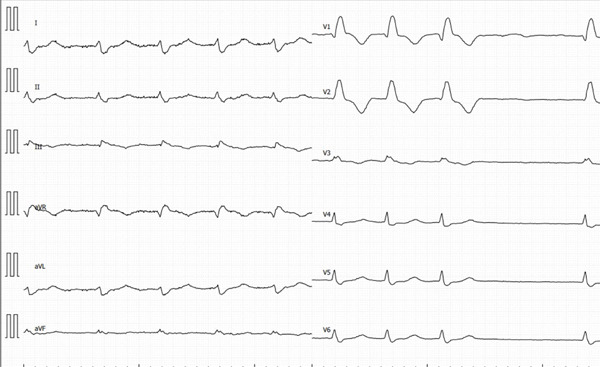
Initial ECG demonstrated atrial fibrillation with a high ventricular rate, complete right bundle branch block, and evidence of anterior myocardial injury, including pathological Q waves and T‐wave inversions in Leads V1–V2.

The initial ECG (Figure 1) showed AF with rapid ventricular response and signs consistent with septal myocardial infarction, such as the presence of right bundle branch block, pathological Q waves, and T‐changes in the septal leads (V1–V2).

### 2.1. Clinical Findings and Diagnostic Workup

On presentation, the patient reported a persistent right arm pain and chest pressure since the previous afternoon, accompanied by exertional dyspnea. She denied nausea, vomiting, and radiation of pain to the left arm, abdomen, or back. No signs of infection, fever, cough, or expectoration were noted. Urination was normal, and there were no episodes of diarrhea.

Upon examination, the patient was alert, oriented, and cooperative. Physical examination revealed the following:

- Pupils: mid‐sized, equal, and reactive to light.
- Right hand: slightly weaker grip.
- Mucous membranes: dry
- Cardiac: irregular heart rhythm (AF), tachycardia, and loud holosystolic murmur.
- Pulmonary: clear bilateral vesicular breath sounds, no additional sounds
- Abdomen: soft, nontender, no guarding, active bowel sounds in all quadrants.
- Extremities: intact perfusion, motor and sensory function, no leg edema, no signs of thrombosis, and a very dry skin.

**Figure 2:**
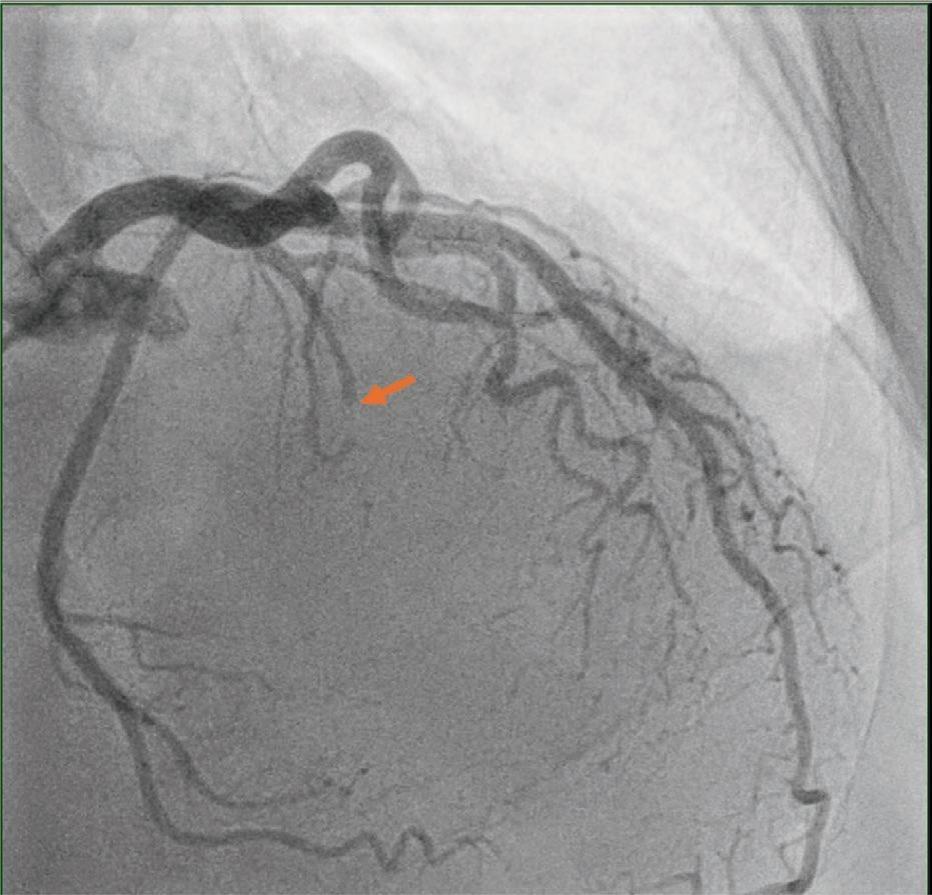
Coronary angiogram in the right anterior oblique (RAO) cranial projection showing an acute obstruction of the first septal branch of the left anterior descending coronary artery.

Initial laboratory investigations revealed an elevated troponin level of 1096 pg/mL. A coronary angiography demonstrated a coronary single‐vessel disease with occlusion of a branch of the first septal perforator (possible embolism vs. congenital anomaly) (Figure 2). The right coronary artery was small and unremarkable.

### 2.2. Transesophageal Echocardiography (TEE) and Cardiac MRI Findings

**Figure 3:**
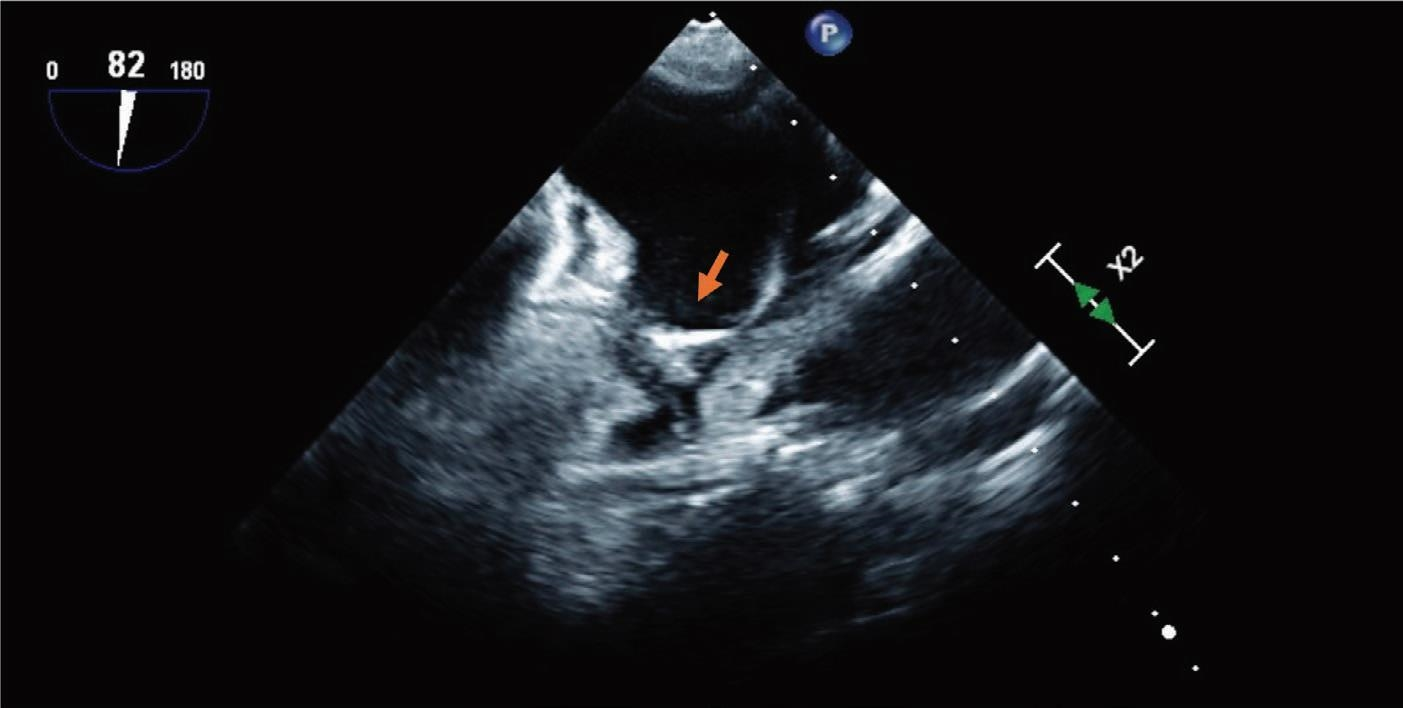
Transesophageal echocardiogram focusing on the left atrial appendage (LAA), showing evidence of thrombus formation.

TEE showed spontaneous echocardiographic contrast in the LAA (Grades II–III) with “sludge formation” and a thrombus at the apex of the superior lobe of the LAA (Figure 3). Other notable findings included the following:

- AV: mild sclerosis.
- MV: mild mitral regurgitation.
- TV: moderate to severe tricuspid regurgitation (atrial functional TI).
- sPAP: 35 mmHg.
- Severe aortic sclerosis.
- No vegetations on valves.
- Moderately dilated right ventricle.
- Left ventricular ejection fraction (LVEF) is approximately 50%.

**Figure 4:**
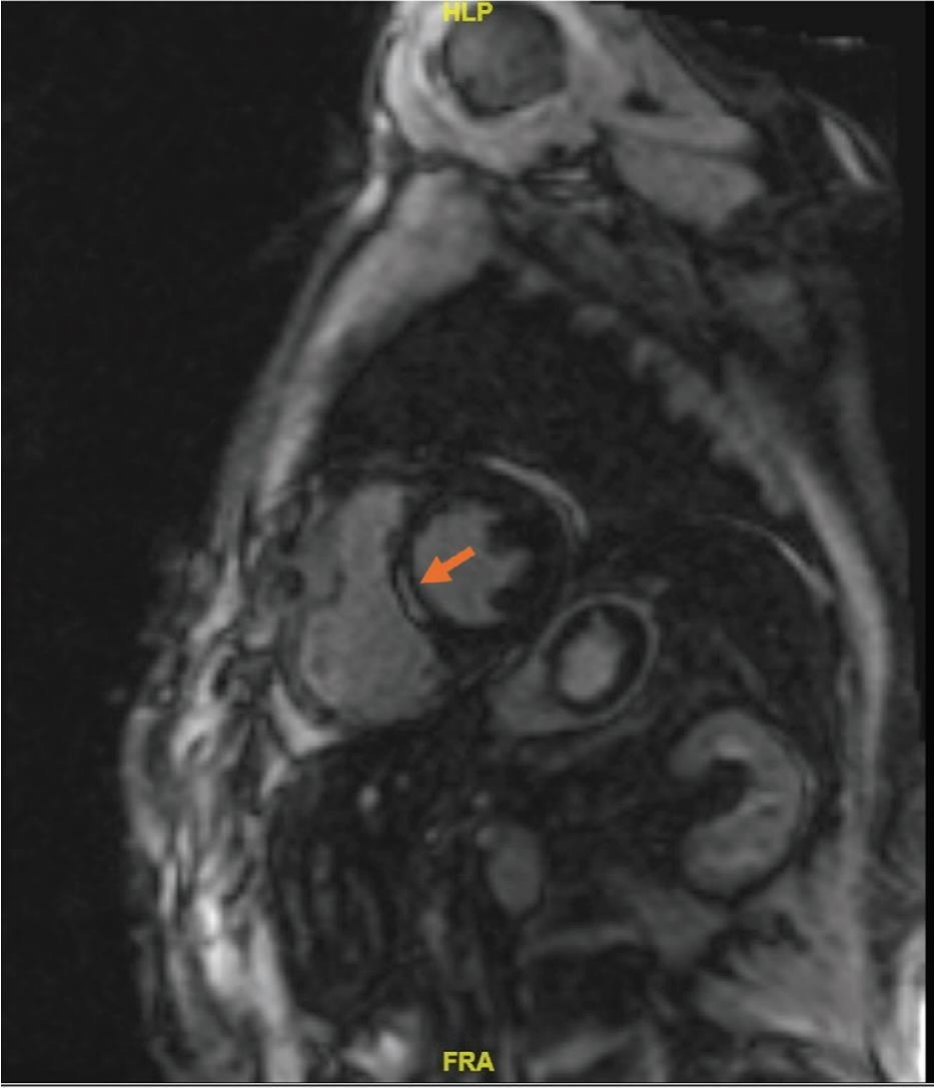
Cardiac magnetic resonance imaging MRI shows isolated late enhancement in the area of the interventricular septum, typically observed on T1‐weighted images.

Cardiac MRI confirmed the following anatomical findings:

- No intraventricular thrombus.
- Basal pericardial effusion up to 5 mm.
- Septal akinesia in the basal region.
- LVEF reduced to 40%.
- Delayed enhancement indicating transmural signal increase in the basal anteroseptal and inferoseptal regions, correlating with septal akinesia (Figure 4).

## 3. Discussion

Several case reports in the literature have described myocardial infarction resulting from coronary artery embolism in the setting of AF [8]. Shibata et al. analyzed 1776 acute MI cases and identified coronary embolism in 2.9%, most commonly linked to AF. These patients had fewer traditional risk factors for coronary artery disease and showed notable benefit from anticoagulation during follow‐up [9].

This case illustrates a rare presentation of an isolated thrombotic occlusion of the septal branch leading to a septal MI, likely secondary to an LAA thrombus in the setting of untreated AF. The patient′s intolerance to standard anticoagulation and subsequent decision to discontinue therapy underscores the importance of patient education and adherence to medical recommendations in managing AF. The echocardiographic and MRI findings were crucial in diagnosing the septal MI and assessing the extent of cardiac involvement.

## 4. Conclusion

Effective management of AF requires adherence to anticoagulation therapy to prevent thromboembolic complications. This case highlights the consequences of discontinuing anticoagulation and the importance of comprehensive patient education and follow‐up. Further studies are needed to explore alternative anticoagulation strategies for patients with medication intolerances.

## Funding

This study was supported by Projekt DEAL, which enabled and organized open access funding.

## Consent

Written informed consent was obtained from the patient for the publication of this manuscript.

## Conflicts of Interest

The authors declare no conflicts of interest.

## Data Availability

The data that support the findings of this study are available from the corresponding author upon reasonable request.
